# Association Between Maternal Exposure to SO_2_ and Congenital Ear Malformations in Offspring: A Population-Based Case-Control Study in Liaoning Province, China

**DOI:** 10.3389/ijph.2022.1604945

**Published:** 2022-07-07

**Authors:** Wei-Jun Yu, Na Li, Ting-Ting Gong, Jia-Yu Zhang, Yu-Ting Jiang, Yu-Hong Zhao, Yan-Hong Huang, Jing Li, Shu Liu, Yan-Ling Chen, Li-Li Li, Cheng-Zhi Jiang, Zong-Jiao Chen, Qi-Jun Wu

**Affiliations:** ^1^ Department of Clinical Epidemiology, Shengjing Hospital of China Medical University, Shenyang, China; ^2^ Institute for Prevention and Control of Infection and Infectious Diseases, Liaoning Provincial Center for Disease Control and Prevention, Shenyang, China; ^3^ Department of Obstetrics and Gynecology, Shengjing Hospital of China Medical University, Shenyang, China; ^4^ Department of Ophthalmology, Shenyang Women’s and Children’s Hospital, Shenyang, China; ^5^ Department of Science and Education, Shenyang Maternity and Child Health Hospital, Shenyang, China; ^6^ Department of Atmospheric Environment Monitoring, Liaoning Eco-environmental Monitoring Center, Shenyang, China; ^7^ Office of Institution, Liaoning Women and Children’s Health Hospital, Shenyang, China; ^8^ Department of Children’s Health Prevention, Shenyang Maternity and Child Health Hospital, Shenyang, China; ^9^ School of Environmental and Chemical Engineering, Shenyang Ligong University, Shenyang, China

**Keywords:** air pollution, congenital ear malformations, microtia, risk, sulfur dioxide

## Abstract

**Objectives:** To examine associations between maternal sulfur dioxide (SO_2_) exposure and congenital ear malformations risk in offspring.

**Methods:** We surveyed 1676 cases with congenital ear malformations and 7950 controls from the Maternal and Child Health Certificate Registry of Liaoning Province between 2010 and 2015. SO_2_ concentrations were obtained from the Municipal Environment Protection Bureau of Liaoning Province. Multivariable logistic regression models and Restricted cubic splines (RCS) model were used to assess the aforementioned association.

**Results:** There were significant associations between maternal SO_2_ exposure and congenital ear malformations risk during the 3 months before conception (OR _Q4 vs. Q1_ = 1.93, 95% CI = 1.43–2.59) and the 3 months after conception (OR _Q4 vs. Q1_ = 1.63, 95% CI = 1.22–2.18). Similar results were obtained in the analysis of single-month exposure windows, except for the third month before conception and the third month after conception. Moreover, these findings were broadly consistent across subgroups and robust in sensitivity analyses. There were non-linear dose-response associations between SO_2_ exposure and congenital ear malformations based on restricted cubic spline model analysis.

**Conclusion:** Maternal SO_2_ exposure is associated with increased congenital ear malformations risk in offspring.

## Introduction

Congenital ear malformations are defined as deformities caused by congenital embryonic developmental disorders. Congenital ear malformations are comprised of microtia, including anotia, and other malformations of the external ear that exclude microtia and anotia. Among these, the main type of congenital ear malformations is microtia [1].

Globally, the prevalence of microtia varies greatly, ranging from 0.83/10,000 to 17.4/10,000 [2]. Based on the China Maternal and Child Health Monitoring and Annual Report, the incidence of microtia in China in 2018 was 2.99 per 10,000 cases [3]. The incidence of congenital ear malformations remains high, which creates great physiological and psychological obstacles and economic burdens to patients. However, no clear cause and mechanism have been found for this disease. Moreover, ear malformations are a multifactorial disease, which may be caused by environmental and genetic factors, as well as interactions between these factors [4, 5]. It has been reported that more than 2 million premature deaths each year are attributed to air pollution, and 91% of the world’s population live in environments where air quality does not meet the standards set by the World Health Organization [6].

In 2002, Ritz et al. [7] first discovered the association between ambient air pollution and congenital malformations, which has since been confirmed by numerous researchers [8–13]. Although evidence from *in vivo* and *in vitro* studies [14–18] seem to indicate that air pollution exposure during pregnancy causes congenital ear malformations, evidence from epidemiological studies remain limited.

Liaoning Province is one of the important old industrial bases in China. Presently, the province has 39 major industries, 197 medium industries, and more than 500 small industries, making it one of the provinces with the most complete industrial profile in China. The rapid development of industries greatly affects the atmospheric environment. The 2016 China Environmental Bulletin, which has been issued by the Ministry of Ecology and Environment of the People’s Republic of China, stated that among China’s 338 prefecture-level and higher cities, 254 of them exceed environmental air quality standards, accounting for 75.1% of cities (http://www.mee.gov.cn), and the sulfur dioxide (SO_2_) concentration ranged from 3 to 88 μm/m^3^, with an average of 22 μm/m^3^. The average annual SO_2_ concentration is 34 μm/m^3^ in Liaoning Province, which is much higher than the national average. In 2014, the annual population-weighted-average value of SO_2_ in China was 34.1 μm/m^3^, and it was 96.7% higher in northern China than that in southern China [19]. Thus, our study seeks to expand the etiological research of congenital ear malformations. The purpose of this case-control study is to estimate the association between maternal SO_2_ exposure and congenital ear malformations risk in offspring during a crucial period.

## Methods

### Study Population and Data Source

The study population was comprised of offspring with congenital ear malformations and healthy infants from 1 January 2010 to 31 December 2015. The study population was recruited from the Maternal and Child Health Certificate Registry of Liaoning Province, which is managed by Liaoning Women and Children’s Health Hospital. Details on the registry have been previously published [20, 21]. In brief, the registry is an active hospital-based monitoring system that is maintained in accordance with the national monitoring program. All 31 provinces in China have launched the active monitoring system. Similar to other provinces, Liaoning Province submits data to a national database maintained by the China Birth Defects Monitoring Network [22]. All 14 major cities in Liaoning Province (Shenyang, Dalian, Anshan, Fushun, Benxi, Dandong, Jinzhou, Yingkou, Fuxin, Liaoyang, Panjin, Tieling, Chaoyang, and Huludao) were covered by the Maternal and Child Health Certificate Registry. Data on congenital malformations were provided by Maternal and Child Health Certificate Registries in 14 cities of Liaoning Province and collected from Liaoning Women and Children’s Health Hospital [22]. During the study period from 2010 to 2015, approximately 6,000 cases of congenital malformations were reported each year from all maternity units in the province [23].

### Cases and Controls

Cases with congenital ear malformations (*n* = 1676) were registered in the Maternal and Child Health Certificate Registry of Liaoning Province and classified as live births, stillbirths, or pregnancy terminations after prenatal diagnosis of congenital anomalies that were born or terminated from 2010 to 2015. According to the International Classification of Diseases, 10th Revision, Clinical Modifications [ICD-10-CM], congenital ear malformations include two major malformations: microtia (including anotia; n = 361; ICD10:Q17.2, Q16.0) and other external ear malformations (except for microtia and anotia; n = 1315; ICD10:Q17).

Controls were selected as previously described [20]. In brief, we divided Liaoning Province into southeastern, western, and central regions according to geographical features. Thereafter, five cities from the three regions (southeastern region: Dalian; western region: Fuxin, Chaoyang, and Huludao; central region: Shenyang) were representatively selected based on the degree of air pollution and socio-economic characteristics. The control group was a random sampling of 1.5% of live births without congenital ear malformations from the five cities in the three regions by random birth-year sampling, which was not case-related (mismatched). Subjects with missing or implausible covariate information were excluded and not included in the final analysis. We conducted this study according to national and local regulations. The Institutional Review Board of Liaoning Women and Children’s Health Hospital reviewed and approved the study protocol, and the study was conducted in compliance with local and national regulations.

### Data Collection and Quality Control

The data collection process was carried out as previously described [21]. In brief, each newborn or terminated fetus was inspected by an experienced obstetric or pediatric specialist after birth. Cases were registered and coded based on the International Classification of Diseases, 10th Edition (ICD10). For cases of suspected congenital ear malformations that were diagnosed by prenatal ultrasonography, we examined newborns or fetuses again after postpartum check or pregnancy termination. Cases were diagnosed in all monitored hospitals from the 14 cities in Liaoning Province, and experienced obstetric or pediatric specialists immediately interviewed the newborn’s mother in order to complete the Birth Defects Registration Form that was used to gather data, including information on maternal and child demographics, diagnosis of congenital defects, history of early pregnancy, and family history. Forms were submitted to the local Maternal and Child Healthcare Institution in the 14 cities, which submitted forms to the Liaoning Women and Children’s Health Hospital. Data were retrospectively verified by a team of experienced genetics and pediatrics specialists [24].

The data quality control was carried out as previously described [24]. Based on the Chinese Maternal and Child Health Surveillance Workbook, specialists from monitoring institutions at all levels diagnosed the disease, and collected and checked the data, as well as the medical records to ensure the high quality of data. To distinguish between inadequacies and inaccuracies, the experts also carried out an independent retrospective investigation [24].

### Exposure Assessment

**FIGURE 1:**
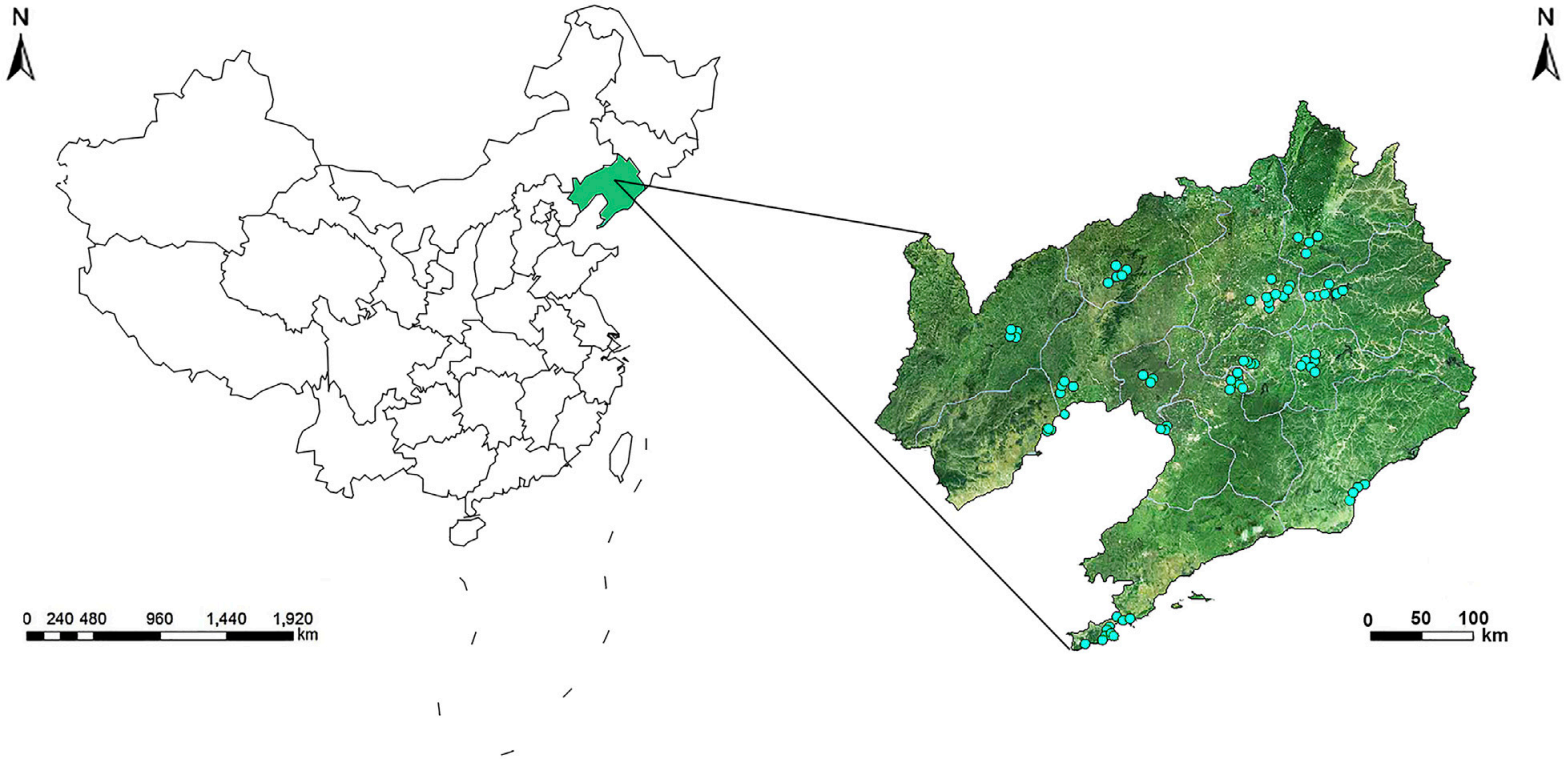
Geographic locations of air monitoring stations in 14 cities in Liaoning Province, China (Association between maternal exposure to SO_2_ and congenital ear malformations in offspring: a population-based case-control study in Liaoning Province, China, 2010–2015).

The environmental air pollutant monitoring station network in Liaoning Province is comprised of 77 monitoring stations in 14 cities (two of which served as controls), and this network assessed air pollutant exposure (except for the two controls) (Figure 1). The 77 air pollutant monitoring stations in Liaoning Province were mainly located in urban areas, where they covered residential areas and represented air pollutant levels in the region. According to the Chinese government’s ambient air quality standards from the China National Environmental Monitoring Centre (CNEMC, http://www.cnemc.cn), all monitoring stations in the 14 cities detected and reported air pollutant concentrations and submitted these data to the Environment Protection Bureau. The exposure levels were assessed using the mean SO_2_ concentration at each monitoring station in the mother’s city of residence, and then these values were used to calculate the monthly mean SO_2_ concentration (Supplementary Figure S1). Monthly mean SO_2_ concentration exposure levels were assigned to each newborn after averaging concentrations from all air pollutant monitoring stations recorded by the Birth Defects Registration Form during the 3 months before conception and the 3 months after conception. The gestational age of cases and controls was estimated according to the due date provided by the obstetrician. We used the date of the last menstrual period to determine the first month of pregnancy. If the date of the last menstrual period occurred in the first half of the month, then this month was considered as the first month of conception. Otherwise, it was considered as the first month before conception.

### Covariates

According to the change in the estimate criterion (10%) [25], we selectively adjusted the corresponding covariates in the model. In the final model, the covariates were maternal age (two categories: <30, ≥30 years), season of conception (four categories: spring [March–May], summer [June–August], fall [September–November], and winter [December–February]), gravidity (two categories: 1, ≥2), parity (two categories: 1, ≥2), maternal education (four categories: elementary school or less, middle school, high school, and college or above), and maternal NO_2_ and PM_10_ exposure levels.

### Statistical Analysis

Congenital ear malformations were treated as a dichotomous category and analyzed as a dependent variable. From the third month before conception to the third month after conception, the SO_2_ exposure concentration was computed as a continuous independent variable and a categorical independent variable based on quartiles of distribution in controls. Characteristics of the categorical variables in cases and controls were presented as frequencies and percentages, and they were compared using the Chi-square test. Means, standard deviations (SD), medians, and percentiles were used to characterize the monthly mean ambient SO_2_ concentration in the 14 cities in Liaoning Province. Spearman’s rank correlation was applied to analyze the correlation between three pollutants (SO_2_, NO_2_, and PM_10_). Odds ratios (ORs) and 95% confidence intervals (CIs) were calculated by multivariable logistic regression analysis. We also examined the associations between maternal SO_2_ exposure, congenital ear malformations, and subgroups in offspring. Specifically, SO_2_ exposure concentrations were divided into quartiles (determined from controls), and the other quartiles (second, third, and fourth) were compared to a reference (the first quartile) to calculate ORs. We conducted linear tests using the median values of SO_2_ exposure in each category as a continuous variable and congenital ear malformations as the response variable. Additionally, ORs in both per 1-standard deviation (SD) and per 10 μm/m^3^ increment were also reported. In our analysis, we respectively fitted single-pollutant (SO_2_ and the aforementioned covariates) and multi-pollutant models (SO_2_+NO_2_; SO_2_+PM_10_; SO_2_+NO_2_+PM_10_) to assess the effects of different pollutants. Furthermore, subgroup analyses were used to investigate the associations between SO_2_ exposure, congenital ear malformations, and maternal age (<30, ≥30 years) using stratification. We added a multiplicative interaction in the regression model between exposure and age to measure the potential interactions.

We performed a sensitivity analysis using propensity score matching (PSM) analysis, which can reduce baseline group differences. Cases with congenital ear malformations (*n* = 1676) and controls (*n* = 7950) were matched using 1:1 nearest-neighbor matching with a caliper width equal to 0.2 of SD of the logit of propensity scores. And propensity scores were estimated by multivariable logistic regression model, with adjusting for maternal age, season of conception, gravidity, parity, and maternal education. If a case subject could not be matched to any control subject, then the case subject was excluded. Absolute standardized differences (ASD) were used to confirm the balance of covariates between cases and matched control individuals [26]. An ASD of less than 10% was considered a negligible difference between cases and controls.

For associations in the multivariable logistic regression analysis, we further used a restricted cubic splines (RCS) model to assess their shapes of dose-response association [27]. Nonlinear associations were modeled by penalized cubic splines with 3 equally spaced knots. For the first and the second months after conception, the minimum value was defined as the referent value by default in the SAS macro “%RCS_Reg” [27]. The 5th percentile value was defined as the reference in the other six exposure windows.

All statistical analyses were conducted using SAS software version 9.4 (SAS Institute Inc., Cary, North Carolina, United States). Statistical significance was taken at *p*-values < 0.05 based on two-sided tests.

## Results

The basic characteristics of cases and controls in Liaoning Province are shown in Table 1. Cases of congenital ear malformations (*n* = 1676) and controls (*n* = 7950) were included in our analysis. Compared with controls, the number of males was greater (57.0%), the gestational age was shorter (<37 weeks, 6.1%), the birth weight was lower (<2500 g, 5.3%), and more births occurred within the fall (26.3%) and the winter (20.8%) for cases. Compared with controls, the mothers of cases were younger (<30 years, 64.4%), less educated (high school or less, 66.9%), and had more pregnancies (≥2, 45.2%) and deliveries (≥2, 19.6%).

**TABLE 1:** Characteristics of controls and cases in Liaoning Province, China, 2010–2015 [no.(%)] (Association between maternal exposure to SO_2_ and congenital ear malformations in offspring: a population-based case-control study in Liaoning Province, China, 2010–2015).

Characteristics	Cases	Controls	*p*-Value
Total	1676 (100)	7950 (100)	
Season of conception			<0.001
Spring	443 (26.4)	2106 (26.5)	
Summer	444 (26.5)	2829 (35.6)	
Fall	440 (26.3)	1705 (21.4)	
Winter	349 (20.8)	1310 (16.5)	
Gender of infant			<0.001
Female	720 (43.0)	3927 (49.4)	
Male	956 (57.0)	4023 (50.6)	
Gestational age, weeks			<0.001
<37	102 (6.1)	257 (3.2)	
≥37	1574 (93.9)	7693 (96.8)	
Birth weight, grams			<0.001
<2500	89 (5.3)	174 (2.2)	
2500–<4000	1410 (84.1)	6840 (86.0)	
≥4000	177 (10.6)	936 (11.8)	
Maternal age, years			<0.001
<30	1080 (64.4)	4704 (59.2)	
≥30	596 (35.6)	3246 (40.8)	
Gravidity			<0.001
1	918 (54.8)	5026 (63.2)	
≥2	758 (45.2)	2924 (36.8)	
Parity			<0.001
1	1348 (80.4)	7695 (96.8)	
≥2	328 (19.6)	255 (3.2)	
Maternal education			<0.001
Elementary school or less	68 (4.1)	265 (3.3)	
Middle school	659 (39.3)	2912 (36.6)	
High school	394 (23.5)	1723 (21.7)	
College or above	555 (33.1)	3050 (38.4)	

Table 2 shows the distribution characteristics of the ambient SO_2_ concentration (μg/m³) in the 14 cities in Liaoning Province from 2010 to 2015. The results of our analysis showed that exposure to the mean SO_2_ concentration in cases was slightly equal to that in controls, whereas exposure to the mean NO_2_ concentration and PM_10_ exposure in cases was slightly lower than that in controls during the 3 months before and the 3 months after conception (Table 3). Meanwhile, there were high correlations of SO_2_ and PM_10_ during the 3 months before conception (*r* = 0.71) and the 3 months after conception (*r* = 0.78).

**TABLE 2:** Ambient SO_2_ concentrations (μg/m^3^) in 14 cities in Liaoning province, China, 2010-2015 (Association between maternal exposure to SO_2_ and congenital ear malformations in offspring: a population-based case-control study in Liaoning Province, China, 2010–2015).

Characteristics	Mean ± SD	Range	Median (25tile-75tile)
**Years**			
2010	42 ± 27	136	33 (23–54)
2011	42 ± 31	139	30 (19–60)
2012	39 ± 31	188	29 (16–55)
2013	43 ± 37	252	30 (19–58)
2014	46 ± 35	191	34 (21–64)
2015	40 ± 33	191	28 (15–58)
**Cities**			
Shenyang	69 ± 59	246	41 (27–102)
Dalian	34 ± 28	108	22 (11–59)
Anshan	51 ± 43	154	28 (17–86)
Fushun	38 ± 22	83	31 (21–51)
Benxi	51 ± 38	133	41 (19–78)
Dandong	36 ± 28	92	20 (15–61)
Jinzhou	42 ± 29	109	31 (22–55)
Yingkou	31 ± 22	78	22 (14–47)
Fuxin	47 ± 22	91	41 (30–60)
Liaoyang	47 ± 27	123	38 (28–61)
Panjin	25 ± 12	55	22 (17–29)
Tieling	31 ± 20	84	25 (15–42)
Chaoyang	39 ± 26	99	29 (18–58)
Huludao	46 ± 28	103	35 (24–65)

Abbreviations: SD, standard deviation; tile, percentile.

**TABLE 3:** The distribution and correlation of air pollutants’ mean level during the 3 months before conception and the 3 months after conception (Association between maternal exposure to SO_2_ and congenital ear malformations in offspring: a population-based case-control study in Liaoning Province, China, 2010–2015).

Air pollutant (μg/m^3^)	Exposure window	Cases (*n* = 1676)		Controls (*n* = 7950)		Correlation coefficients		
		Mean ± SD	Median (25tile-75tile)	Mean ± SD	Median (25tile-75tile)	SO_2_	NO_2_	PM_10_
SO_2_	The 3 months before conception	47 ± 38	35 (22–63)	48 ± 41	34 (23–59)	1	0.50	0.71
NO_2_		34 ± 10	34 (28–41)	37 ± 9	36 (31–42)		1	0.50
PM_10_		88 ± 24	86 (71–101)	93 ± 27	89 (75–105)			1
SO_2_	The 3 months after conception	47 ± 36	35 (23–63)	47 ± 42	30 (21–65)	1	0.56	0.78
NO_2_		35 ± 10	34 (28–41)	37 ± 9	35 (31–42)		1	0.53
PM_10_		88 ± 25	86 (70–101)	91 ± 28	87 (68–106)			1

Abbreviations: SD, standard deviation; tile, percentile.

There was a significant association between maternal SO_2_ exposure and congenital ear malformations risk during the 3 months before conception and the 3 months after conception. The deleterious effects of maternal SO_2_ exposure on congenital ear malformations remained strong during the exposure window of each single month, except for the third month before conception and the third month after conception (Table 4). When investigating congenital ear malformations subtypes, the higher effect estimates of SO_2_ were observed among conception in external ear malformations compared to in microtia during almost the entire exposure window (Supplementary Table S1). The risk of congenital ear malformations to SO_2_ exposure during the 3 months before conception and the 3 months after conception was greater for younger mothers (age <30 years old) (Supplementary Table S2).

**TABLE 4:** Associations between maternal exposure to ambient SO_2_ during various exposure windows and the risk of congenital ear malformations in offspring (Association between maternal exposure to SO_2_ and congenital ear malformations in offspring: a population-based case-control study in Liaoning Province, China, 2010–2015).

Quartile of SO_2_ level^a^	No. of cases	No. of controls	Unadjusted OR (95% CI)	Model 1^b^ (95% CI)	Model 2^c^ (95% CI)	Model 3^d^ (95% CI)	Model 4^e^ (95% CI)
Pre-conception, 0–1 month							
<19	401	1983	1.00 (ref)	1.00 (ref)	1.00 (ref)	1.00 (ref)	1.00 (ref)
19 to <29	337	1931	0.86 (0.74–1.01)	0.83 (0.70–0.98)	0.83 (0.70–0.98)	0.89 (0.75–1.06)	0.85 (0.72–1.02)
29 to <52	432	2048	1.04 (0.90–1.21)	0.87 (0.73–1.05)	1.02 (0.84–1.23)	1.07 (0.88–1.29)	1.09 (0.90–1.33)
≥52	506	1988	1.26 (1.09–1.46)	0.94 (0.76–1.17)	1.48 (1.17–1.87)	1.27 (1.01–1.60)	1.61 (1.27–2.05)
*P*_trend_			<0.001	0.803	<0.001	0.012	<0.001
Per 1-SD increase			1.03 (0.98–1.09)	0.83 (0.76–0.91)	1.10 (1.00–1.20)	1.06 (0.95–1.18)	1.24 (1.11–1.38)
Per 10 μg/m^3^ increase			1.01 (1.00–1.02)	0.96 (0.94–0.98)	1.02 (1.00–1.04)	1.01 (0.99–1.04)	1.05 (1.02–1.07)
Pre-conception, 1–2 months							
<19	369	1713	1.00 (ref)	1.00 (ref)	1.00 (ref)	1.00 (ref)	1.00 (ref)
19 to <31	404	2225	0.84 (0.72–0.98)	0.84 (0.71–0.99)	0.88 (0.75–1.05)	0.91 (0.77–1.08)	0.92 (0.77–1.09)
31 to <54	399	1949	0.95 (0.81–1.11)	0.98 (0.81–1.18)	1.17 (0.96–1.42)	1.25 (1.03–1.52)	1.28 (1.05–1.58)
≥54	504	2063	1.13 (0.98–1.32)	1.02 (0.81–1.28)	1.63 (1.27–2.08)	1.43 (1.13–1.83)	1.83 (1.42–2.38)
*P*_trend_			0.001	0.476	<0.001	0.003	<0.001
Per 1-SD increase			1.00 (0.95–1.05)	0.87 (0.80–0.94)	1.11 (1.02–1.20)	1.07 (0.97–1.18)	1.23 (1.11–1.36)
Per 10 μg/m^3^ increase			1.00 (0.99–1.01)	0.97 (0.95–0.99)	1.02 (1.00–1.04)	1.01 (0.99–1.04)	1.05 (1.02–1.07)
Pre-conception, 2–3 months							
<23	511	1728	1.00 (ref)	1.00 (ref)	1.00 (ref)	1.00 (ref)	1.00 (ref)
23 to <35	363	2186	0.56 (0.48–0.65)	0.55 (0.47–0.65)	0.61 (0.51–0.72)	0.59 (0.50–0.70)	0.61 (0.52–0.73)
35 to <67	388	1959	0.67 (0.58–0.78)	0.57 (0.47–0.70)	0.79 (0.64–0.96)	0.66 (0.54–0.80)	0.80 (0.65–0.98)
≥67	414	2077	0.67 (0.58–0.78)	0.47 (0.37–0.60)	0.87 (0.68–1.11)	0.63 (0.49–0.81)	0.90 (0.69–1.17)
*P*_trend_			0.005	<0.001	0.586	0.050	0.351
Per 1-SD increase			0.90 (0.85–0.95)	0.78 (0.72–0.85)	1.02 (0.93–1.12)	0.91 (0.82–1.01)	1.07 (0.96–1.20)
Per 10 μg/m^3^ increase			0.98 (0.97–0.99)	0.95 (0.93–0.97)	1.00 (0.99–1.02)	0.98 (0.96–1.00)	1.02 (0.99–1.04)
Pre-conception, 0–3 months							
<23	466	1900	1.00 (ref)	1.00 (ref)	1.00 (ref)	1.00 (ref)	1.00 (ref)
23 to <34	343	2080	0.67 (0.58–0.78)	0.59 (0.50–0.70)	0.79 (0.66–0.93)	0.74 (0.62–0.88)	0.87 (0.73–1.04)
34 to <59	401	1981	0.83 (0.71–0.96)	0.71 (0.58–0.87)	1.03 (0.83–1.28)	0.98 (0.79–1.21)	1.21 (0.96–1.51)
≥59	466	1989	0.96 (0.83–1.10)	0.66 (0.51–0.84)	1.40 (1.07–1.83)	1.27 (0.96–1.68)	1.93 (1.43–2.59)
*P*_trend_			0.121	0.100	<0.001	<0.001	<0.001
Per 1-SD increase			0.98 (0.92–1.03)	0.81 (0.74–0.88)	1.07 (0.98–1.17)	1.13 (1.01–1.26)	1.36 (1.20–1.53)
Per 10 μg/m^3^ increase			0.99 (0.98–1.01)	0.95 (0.93–0.97)	1.02 (1.00–1.04)	1.03 (1.00–1.06)	1.08 (1.05–1.11)
Post-conception, 0–1 month							
<17	322	1896	1.00 (ref)	1.00 (ref)	1.00 (ref)	1.00 (ref)	1.00 (ref)
17 to <29	431	2051	1.24 (1.06–1.45)	1.15 (0.97–1.37)	1.11 (0.93–1.31)	1.29 (1.09–1.53)	1.17 (0.98–1.39)
29 to <52	411	1958	1.24 (1.06–1.45)	0.95 (0.78–1.15)	1.11 (0.91–1.35)	1.26 (1.03–1.55)	1.23 (1.00–1.52)
≥52	512	2045	1.47 (1.27–1.72)	0.89 (0.71–1.13)	1.51 (1.19–1.92)	1.44 (1.12–1.85)	1.77 (1.37–2.29)
*P*_trend_			<0.001	0.199	<0.001	0.028	<0.001
Per 1-SD increase			1.01 (0.96–1.07)	0.76 (0.69–0.83)	1.02 (0.93–1.12)	0.96 (0.85–1.08)	1.13 (1.01–1.27)
Per 10 μg/m^3^ increase			1.00 (0.99–1.01)	0.94 (0.92–0.96)	1.00 (0.98–1.03)	0.99 (0.97–1.02)	1.03 (1.00–1.05)
Post-conception, 1–2 months							
<17	318	1960	1.00 (ref)	1.00 (ref)	1.00 (ref)	1.00 (ref)	1.00 (ref)
17 to <29	427	1866	1.41 (1.20–1.65)	1.30 (1.10–1.54)	1.27 (1.07–1.51)	1.44 (1.21–1.72)	1.31 (1.10–1.57)
29 to <58	436	2087	1.29 (1.10–1.51)	0.96 (0.79–1.17)	1.24 (1.02–1.51)	1.22 (1.00–1.49)	1.32 (1.08–1.62)
≥58	495	2037	1.50 (1.29–1.75)	0.80 (0.63–1.02)	1.34 (1.04–1.73)	1.22 (0.94–1.60)	1.49 (1.14–1.95)
*P*_trend_			<0.001	0.007	0.111	0.703	0.039
Per 1-SD increase			1.00 (0.94–1.05)	0.75 (0.69–0.82)	0.99 (0.90–1.09)	0.89 (0.79–0.99)	1.03 (0.92–1.16)
Per 10 μg/m^3^ increase			1.00 (0.99–1.01)	0.94 (0.92–0.96)	1.00 (0.98–1.02)	0.97 (0.95–1.00)	1.01 (0.98–1.03)
Post-conception, 2–3 months							
<18	347	1932	1.00 (ref)	1.00 (ref)	1.00 (ref)	1.00 (ref)	1.00 (ref)
18 to <32	433	1906	1.27 (1.08–1.48)	1.12 (0.94–1.33)	1.14 (0.96–1.36)	1.25 (1.05–1.49)	1.20 (1.01–1.43)
32 to <66	485	2105	1.28 (1.10–1.49)	0.90 (0.74–1.09)	1.17 (0.96–1.44)	1.15 (0.93–1.41)	1.28 (1.04–1.59)
≥66	411	2007	1.14 (0.98–1.33)	0.56 (0.43–0.72)	1.00 (0.76–1.30)	0.85 (0.65–1.11)	1.15 (0.87–1.52)
*P*_trend_			0.599	<0.001	0.427	0.014	0.936
Per 1-SD increase			0.96 (0.91–1.01)	0.70 (0.64–0.77)	0.93 (0.84–1.02)	0.84 (0.75–0.94)	1.00 (0.89–1.13)
Per 10 μg/m^3^ increase			0.99 (0.98–1.00)	0.93 (0.91–0.95)	0.98 (0.96–1.01)	0.96 (0.94–0.99)	1.00 (0.98–1.03)
Post-conception, 0–3 months							
<21	378	2002	1.00 (ref)	1.00 (ref)	1.00 (ref)	1.00 (ref)	1.00 (ref)
21 to <30	345	2028	0.90 (0.77–1.06)	0.81 (0.68–0.96)	0.95 (0.80–1.13)	1.00 (0.84–1.19)	1.05 (0.88–1.25)
30 to <65	549	1892	1.54 (1.33–1.78)	0.98 (0.81–1.19)	1.37 (1.12–1.68)	1.45 (1.17–1.79)	1.64 (1.32–2.03)
≥65	404	2028	1.06 (0.91–1.23)	0.54 (0.42–0.70)	1.19 (0.91–1.55)	1.12 (0.84–1.48)	1.63 (1.22–2.18)
*P*_trend_			0.303	<0.001	0.873	0.292	0.063
Per 1-SD increase			0.99 (0.93–1.04)	0.70 (0.63–0.76)	0.96 (0.87–1.06)	0.98 (0.86–1.11)	1.18 (1.03–1.34)
Per 10 μg/m^3^ increase			1.00 (0.98–1.01)	0.92 (0.90–0.94)	0.99 (0.97–1.01)	1.00 (0.97–1.03)	1.04 (1.01–1.07)

Abbreviations: CI, confidence interval; OR, odds ratios; SD, standard deviation; SO_2_, sulfur dioxide; ref, reference.

a SO_2_ concentrations (μg/m³) are based on the monthly average concentrations, which are then averaged over different exposure windows and analyzed in quartiles (determined from controls).

b Model 1 adjusted for maternal age, season of conception, gravidity, parity and maternal education.

c Model 2 adjusted for covariates in model 1 plus nitrogen dioxide exposure levels during the same period.

d Model 3 adjusted for covariates in model 1 plus particulate matter with an aerodynamic diameter ≤10 μm exposure levels during the same period.

e Model 4 adjusted for covariates in model 1 plus nitrogen dioxide and particulate matter with an aerodynamic diameter ≤10 μm exposure levels during the same period.

In the sensitivity analyses, a more balanced subsample of 767 congenital ear malformations cases and 767 matched controls were generated after 1:1 PSM. As shown in Supplementary Table S3, PSM removed the enormous imbalance in selected covariate distributions. SO_2_ exposure treated as a categorical variable or as a continuous one was strongly associated with the risk of congenital ear malformations during the entire exposure window (Supplementary Table S4).

**FIGURE 2:**
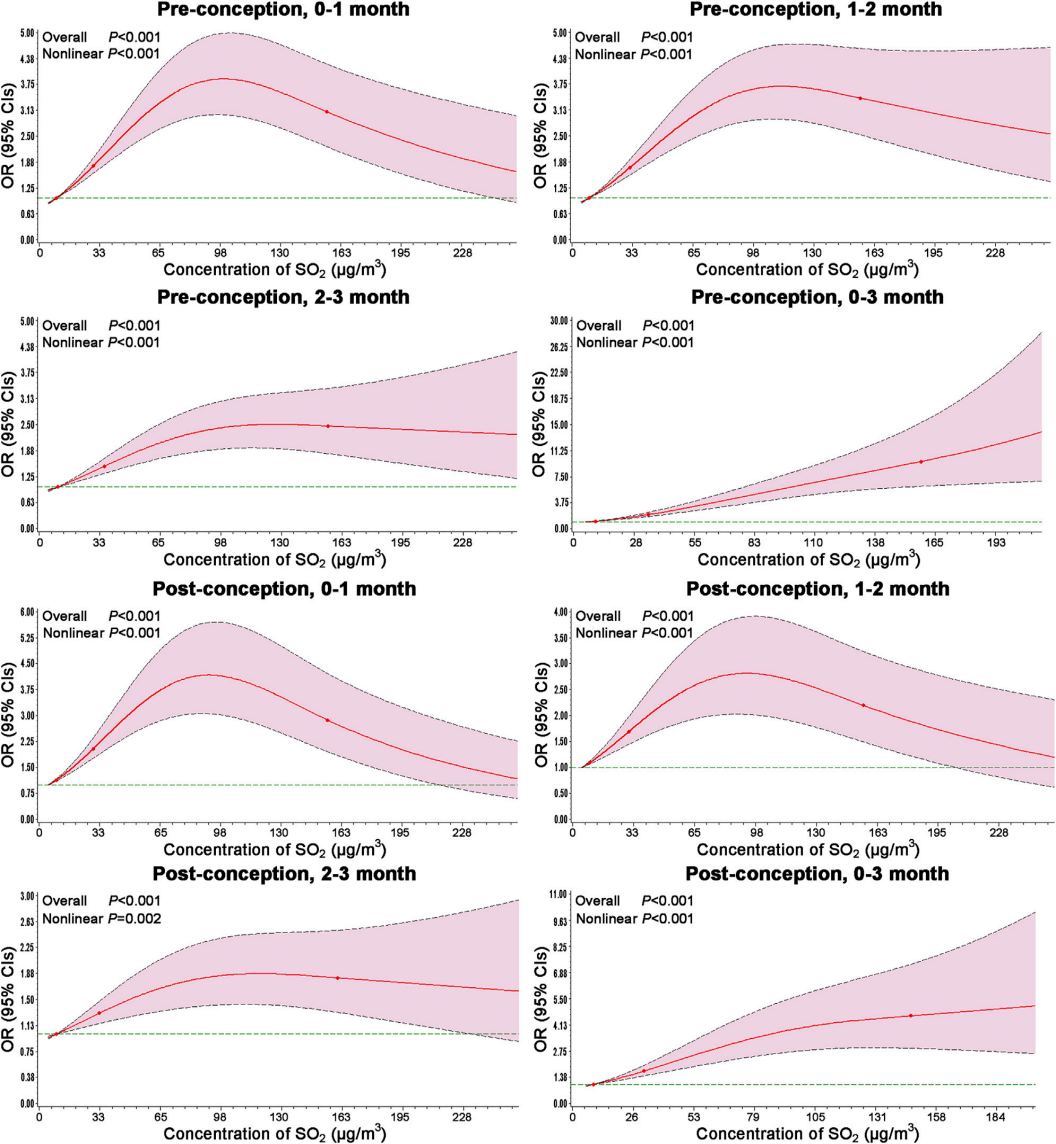
Dose-response associations between ambient SO_2_ exposure and the risk of congenital ear malformations modeled by RCS. Models adjusted for maternal age, season of conception, gravidity, parity, maternal education, NO_2_ and PM_10_ exposure levels during the same period. Odds ratios represented by bold line, and 95% confidence intervals represented by shaded area (Association between maternal exposure to SO_2_ and congenital ear malformations in offspring: a population-based case-control study in Liaoning Province, China, 2010–2015).

The results of RCS models are shown in Figure 2. The risks of congenital ear malformations versus SO_2_ exposure were analyzed by incorporating the selected covariate using the RCS model analyses. And the associations between SO_2_ exposure and congenital ear malformations risk were further confirmed with non-linear dose-response association during the entire exposure window.

## Discussion

This is the first study to examine the association between maternal SO_2_ exposure and congenital ear malformations risk in offspring. Specifically, we observed significant positive associations during the 3 months before conception and the 3 months after conception. And above associations between SO_2_ exposure and congenital ear malformations risk were curvilinear and also remained robust after 1:1 PSM. Moreover, subgroups analyses stratified by ear malformation subtype and maternal age were broadly consistent with the main results.

The exact bio-mechanism by which SO_2_ exposure during conception increases the risk of congenital ear malformations in offspring is still unclear. The auricle develops from the first and the second branchial arches during the embryonic period of 5–9 weeks [28], so it is generally considered to be the joint site of action for the pathological occurrence of congenital microtia [1]. The influencing mechanism behind various risk factors, including air pollution, may be the abnormal migration of cranial neural crest cells that leads to the occurrence of congenital microtia [29]. Evidence from candidate gene and epigenome-wide association studies have suggested that maternal exposure to air pollutants during conception can cause locus-specific changes in methylation, newborn cord blood, and the placenta, particularly in genes involved in cellular responses to oxidative stress, mitochondrial function, inflammation, growth, and early life development [14]. Meanwhile, limited animal experimental studies confirm the association. Research published in 2017 by Calderón-Garcidueñas et al. [16] showed that healthy young dogs in Mexico City exposed to super standard fine particulate matter (PM) and O_3_ showed an association between auditory nuclei dysmorphology and the brainstem auditory evoked potential (BAEP). It is important to note that an epidemiological study they previously conducted in Mexico City in 2011 had estimated this association, as they were conducting this animal experimental study at the time [30]. Given that zebrafish are morphologically similar to hair cells in the human inner ear, zebrafish are widely used to assess ototoxicity. In their most recent study, Rhee et al. [17] used the zebrafish model to assess damage and developmental toxicity of hair cells caused by PM_2.5_ exposure. They observed significant hair cell damage after exposure to PM_2.5_, which was dose-dependent and more severe after prolonged exposure. *In vivo* experiments conducted by Yadav et al. [18] showed that in the presence of urban particles, the number of *Streptococcus pneumoniae* in the nasopharynx of mice increased and spread to the middle ear and lungs, thereby causing pathological changes.

The literature on the association between maternal air pollution exposure and congenital ear malformations risk is sparse. To date, only four studies have focused on this research area [31–34], and they are not directly comparable to the results of this study. Although these studies involved different regions, races, study types, sample sizes, covariate controls, exposure assessment, and statistical analysis methods, they have generated consistent findings in that maternal exposure to air pollution increased the risk of congenital ear malformations. The earliest epidemiological study was a population-based case-control study conducted by Rankin et al. [31] in a northern part of the United Kingdom. They found that eye, ear, face, and neck anomalies were positively non-significantly associated with maternal SO_2_ exposure. Nevertheless, for ear malformations, they failed to provide specific results. Additionally, Pedersen et al. [32] reported that a 10 μg/m^3^ increment in the NO_2_ concentration during the first trimester was positively associated with the risk of ear, face, and neck anomalies after adjusting for parental age, maternal smoking, maternal alcohol consumption, maternal education, and disposable income based on the Danish National Birth Cohort. In 2013, Vinikoor-Imler et al. [33] compiled a state-wide birth cohort from North Carolina, which used a hierarchical Bayesian model to assess maternal fine particulate matter (PM_2.5_) and ozone (O_3_) exposure levels from weeks 3–8 of gestation. Binomial regression model analysis showed that microtia and anotia were positively correlated with maternal PM_2.5_ and O_3_ exposure, but the 95% CI was wide and included the null. Increased odds of microtia and anotia in association with the NO_2_ concentration have been reported in another study conducted in the United States [34]. Previously, the team had investigated the association between traffic-related air pollution and selected congenital anomalies such as neural tube defects, orofacial clefts, gastroschisis [35], and congenital heart defects [36]. Consistent with the results of Rankin et al. [31], we detected congenital malformations through active surveillance across more than 6 years in a relatively large geographic area and analyzed maternal SO_2_ exposure as quartiles using the distribution among the compared population. However, we had a relatively larger sample size and higher SO_2_ exposure level. Furthermore, we adjusted for more covariates. In addition, Pedersen et al. [32] and Padula et al. [34] examined the effects of NO_2_, but neither reported the risk estimates based on the co-pollutant adjusted model. Interestingly, in our study, after adjustment for NO_2_, we observed a significant association between maternal SO_2_ exposure and congenital ear malformations risk, suggesting that this association may be significantly influenced by NO_2_. However, although data for other air pollutants, such as PM_2.5_ and O_3_, may be important for exposure assessment during conception, these data were not available in this study.

Pregnancy is a very critical period that is sensitive to external toxic and harmful substances, including air pollutants. For the attenuating or the masking of association, there is a plausible explanation that the timing of environmental influences for the development of certain congenital malformations is narrow and precise. Congenital malformations mainly occur during the organ-forming period of 3–8 weeks in the first trimester, so the precise window of exposure is crucial in terms of the type of malformations that causes them. For example, the window of ear development is generally 5–8 weeks [37]. The exposure window periods of these four epidemiological studies were different from each other. One study [34] investigated this research topic from the first to the second month of pregnancy, whereas the other [33] focused on the exposure window from weeks 3–8 of pregnancy. Owing to the paucity of epidemiological evidence, it is difficult to arrive at a conclusion at this stage, and further studies are urgently needed.

### Strengths and Limitations

One of the strengths of this study is that it provides original findings on the effects of exposure to ambient SO_2_ during the 3 months before conception and the 3 months after conception and that the SO_2_ concentration is associated with congenital ear malformations risk. The large sample was selected from the Maternal and Child Health Certificate Registry of Liaoning Province from 2010 to 2015, and it included 1676 cases of congenital ear malformations and 7950 controls, which allowed us to investigate the topic. Additionally, maternal SO_2_ exposure (data from the Environment Protection Bureau of 14 cities in Liaoning Province) was assessed in detail. The exposure window included the 3 months before conception and the 3 months after conception to ensure that the strength of the association was fully assessed. Finally, we performed sensitivity and subgroup analyses stratified by congenital ear malformations subtypes and maternal age (<30, ≥30 years), and the results showed stability of associations. The RCS combines quantitative data with the strength of association for the occurrence of outcome, enabling a continuous presentation of the association strength dose-response relationship.

However, there are some limitations, and we suggest interpreting our results with caution. Firstly, the assessment of individual SO_2_ exposure concentrations may have led to exposure misclassification. Because it is difficult to accurately collect individual exposure levels, we used fixed-site monitoring to represent the exposure to pollutants, which inevitably led to bias in exposure assessments. Therefore, to reduce the misclassification of exposure in future studies, it may be necessary to use more sophisticated models to evaluate SO_2_ exposure such as land-use regression models or spatial interpolation models [38]. Secondly, exposure assessment was based on fixed residential addresses, but the residential address of mothers may have changed during conception. For example, the mobility rate was 9% in the northern UK study [39], but it was 3.1% in the Lanzhou study in China [9]. Nevertheless, Lupo et al. [40] have reported that changes in residential addresses during pregnancy had little effect on these assessments. Thirdly, outdoor air pollutant exposure was only captured, but indoor microenvironment air pollutant exposure, such as homes and workplaces, was not, which may have also led to the misclassification of exposure, and the overestimation or underestimation of effects. Fourthly, although the case information of congenital malformations was obtained through the monitoring network and had various quality controls, it was difficult to guarantee absolute consistency in the process of determining and collecting cases due to the differences in diagnosis among different hospitals, and the longest diagnosis time was 7 days after birth, which may have led to the loss of cases. Finally, other unmeasured covariates, such as maternal disease, maternal or paternal smoking, family history of genetic disease, and toxicity exposure during pregnancy, which have been reported in previous studies as possible risk factors for congenital ear malformations [5, 41], may have influenced our results.

### Conclusion

The present study suggests that maternal SO_2_ exposure is associated with increased risks of congenital ear malformations in offspring. Future studies are still needed to confirm or refute these associations and provide scientific evidence for possible public health intervention during the crucial period of ear development.

## Data Availability

Detailed analytical data are stored by the authors and are available on request.

## Abbreviations

CIs, confidence intervals; NO_2_, nitrogen dioxide; ORs, odds ratios; O_3_, ozone; PM, particulate matter; PM_10_, particulate matter with aerodynamic diameter ≤10 μm; PM_2.5_, particulate matter with aerodynamic diameters ≤2.5 μm; PSM, propensity score matching; RCS, restricted cubic splines; SD, standard deviation; SO_2_, sulfur dioxide; Tile, percentile.
