# Supplementary material for: Association Between Maternal Exposure to SO2 and Congenital Ear Malformations in Offspring: A Population-Based Case-Control Study in Liaoning Province, China
Source: Int J Public Health. 2022 Jul 7;67:1604945. doi: 10.3389/ijph.2022.1604945 (PMC9302193; doi:10.3389/ijph.2022.1604945)
Supplement: Supplementary file 1 [file Table1.docx]

**Supplemental Table S1 The associations between ambient SO_2_ exposure (μg/m^3^) and microtia (N=361) and other malformations of external ear (N=1315), subtypes of congenital ear malformations. (Association between maternal exposure to SO_2_ and congenital ear malformations in offspring: a population-based case-control study in Liaoning Province, China, 2010–2015)**

| **Quartile of SO_2_ level^a^** | **No. of controls** | **Microtia** | | | **Other malformations of external ear** | | |
| --- | --- | --- | --- | --- | --- | --- | --- |
|  |  | **No. of cases** | | **Adjusted OR^b^ (95% CI)** | **No. of cases** | | **Adjusted OR^b^ (95% CI)** |
| **Pre-conception, 0–1 month** | | | | | | | |
| <19 | 1983 | 104 | 1.00 (ref) | | 297 | 1.00 (ref) | |
| 19 to <29 | 1931 | 60 | 0.56 (0.39-0.80) | | 277 | 0.94 (0.78-1.14) | |
| 29 to <52 | 2048 | 83 | 0.84 (0.57-1.23) | | 349 | 1.21 (0.97-1.51) | |
| ≥52 | 1988 | 114 | 1.31 (0.83-2.07) | | 392 | 1.78 (1.36-2.32) | |
| Per 1-SD increase |  |  | 1.30 (1.04-1.62) | |  | 1.22 (1.08-1.38) | |
| Per 10 μg/m^3^ increase |  |  | 1.06 (1.01-1.11) | |  | 1.04 (1.02-1.07) | |
| **Pre-conception, 1–2 months** | | | | | | | |
| <19 | 1713 | 100 | 1.00 (ref) | | 270 | 1.00 (ref) | |
| 19 to <31 | 2225 | 75 | 0.61 (0.44-0.86) | | 338 | 1.04 (0.86-1.25) | |
| 31 to <54 | 1949 | 80 | 0.99 (0.66-1.48) | | 310 | 1.35 (1.07-1.69) | |
| ≥54 | 2063 | 106 | 1.26 (0.78-2.07) | | 397 | 1.99 (1.50-2.65) | |
| Per 1-SD increase |  |  | 1.29 (1.05-1.58) | |  | 1.23 (1.10-1.37) | |
| Per 10 μg/m^3^ increase |  |  | 1.06 (1.01-1.10) | |  | 1.04 (1.02-1.07) | |
| **Pre-conception, 2–3 months** | | | | | | | |
| <23 | 1728 | 123 | 1.00 (ref) | | 388 | 1.00 (ref) | |
| 23 to <35 | 2186 | 71 | 0.50 (0.35-0.70) | | 292 | 0.64 (0.53-0.78) | |
| 35 to <67 | 1959 | 82 | 0.71 (0.47-1.07) | | 306 | 0.83 (0.66-1.05) | |
| ≥67 | 2077 | 85 | 0.71 (0.42-1.20) | | 329 | 0.96 (0.72-1.28) | |
| Per 1-SD increase |  |  | 0.93 (0.74-1.17) | |  | 1.10 (0.98-1.25) | |
| Per 10 μg/m^3^ increase |  |  | 0.98 (0.94-1.03) | |  | 1.02 (1.00-1.05) | |
| **Pre-conception, 0–3 months** | | | | | | | |
| <23 | 1900 | 109 | 1.00 (ref) | | 357 | 1.00 (ref) | |
| 23 to <34 | 2080 | 64 | 0.74 (0.51-1.06) | | 279 | 0.92 (0.76-1.12) | |
| 34 to <59 | 1981 | 91 | 1.31 (0.82-2.06) | | 308 | 1.21 (0.94-1.56) | |
| ≥59 | 1989 | 97 | 1.92 (1.06-3.47) | | 371 | 2.04 (1.47-2.84) | |
| Per 1-SD increase |  |  | 1.42 (1.12-1.81) | |  | 1.35 (1.18-1.54) | |
| Per 10 μg/m^3^ increase |  |  | 1.09 (1.03-1.15) | |  | 1.08 (1.04-1.11) | |
| **Post-conception, 0–1 month** | | | | | | | |
| <17 | 1896 | 79 | 1.00 (ref) | | 243 | 1.00 (ref) | |
| 17 to <29 | 2051 | 80 | 0.92 (0.65-1.30) | | 351 | 1.23 (1.02-1.49) | |
| 29 to <52 | 1958 | 90 | 1.23 (0.83-1.84) | | 321 | 1.27 (1.01-1.60) | |
| ≥52 | 2045 | 112 | 1.68 (1.03-2.73) | | 400 | 1.88 (1.41-2.50) | |
| Per 1-SD increase |  |  | 1.09 (0.86-1.38) | |  | 1.15 (1.01-1.31) | |
| Per 10 μg/m^3^ increase |  |  | 1.02 (0.97-1.07) | |  | 1.03 (1.00-1.06) | |
| **Post-conception, 1–2 months** | | | | | | | |
| <17 | 1960 | 71 | 1.00 (ref) | | 247 | 1.00 (ref) | |
| 17 to <29 | 1866 | 90 | 1.33 (0.94-1.88) | | 337 | 1.30 (1.07-1.58) | |
| 29 to <58 | 2087 | 81 | 1.27 (0.83-1.94) | | 355 | 1.37 (1.09-1.71) | |
| ≥58 | 2037 | 119 | 1.80 (1.06-3.08) | | 376 | 1.45 (1.08-1.96) | |
| Per 1-SD increase |  |  | 1.02 (0.82-1.28) | |  | 1.05 (0.93-1.18) | |
| Per 10 μg/m^3^ increase |  |  | 1.01 (0.96-1.05) | |  | 1.01 (0.98-1.04) | |
| **Post-conception, 2–3 months** | | | | | | | |
| <18 | 1932 | 81 | 1.00 (ref) | | 266 | 1.00 (ref) | |
| 18 to <32 | 1906 | 93 | 1.12 (0.79-1.58) | | 340 | 1.22 (1.01-1.48) | |
| 32 to <66 | 2105 | 98 | 1.02 (0.65-1.58) | | 387 | 1.40 (1.11-1.76) | |
| ≥66 | 2007 | 89 | 0.87 (0.50-1.52) | | 322 | 1.29 (0.95-1.75) | |
| Per 1-SD increase |  |  | 0.91 (0.71-1.16) | |  | 1.04 (0.91-1.18) | |
| Per 10 μg/m^3^ increase |  |  | 0.98 (0.93-1.03) | |  | 1.01 (0.98-1.04) | |
| **Post-conception, 0–3 months** | | | | | | | |
| <21 | 2002 | 87 | 1.00 (ref) | | 291 | 1.00 (ref) | |
| 21 to <30 | 2028 | 66 | 0.99 (0.69-1.43) | | 279 | 1.07 (0.87-1.30) | |
| 30 to <65 | 1892 | 121 | 1.65 (1.06-2.55) | | 428 | 1.69 (1.33-2.14) | |
| ≥65 | 2028 | 87 | 1.73 (0.97-3.06) | | 317 | 1.68 (1.22-2.32) | |
| Per 1-SD increase |  |  | 1.23 (0.93-1.62) | |  | 1.19 (1.03-1.37) | |
| Per 10 μg/m^3^ increase |  |  | 1.05 (0.98-1.12) | |  | 1.04 (1.01-1.08) | |

Abbreviations: CI, confidence interval; OR, odds ratios; SD, standard deviation; SO_2_,sulfur dioxide; ref, reference.

^a^ SO_2_ concentrations (μg/m³) are based on the monthly average concentrations, which are then averaged over different exposure windows and analyzed in quartiles (determined from controls).

^b^ Logistic regression analysis adjusting for maternal age, season of conception, gravidity, parity, maternal education and maternal nitrogen dioxide and particulate matter with an aerodynamic diameter ≤ 10 μm exposures during the same exposure window.

**Supplemental Table S2 The maternal age stratification (<30, ≥30 years) subgroups were analyzed for the associations between ambient SO_2_ exposure (μg/m^3^) with controls and cases. (Association between maternal exposure to SO_2_ and congenital ear malformations in offspring: a population-based case-control study in Liaoning Province, China, 2010–2015)**

| **Quartile of SO_2_ level^a^** | **<30 years (5784)** | | | **Quartile of SO_2_ level^a^** | **≥30 years (3842)** | | | **P for**  **interaction** |
| --- | --- | --- | --- | --- | --- | --- | --- | --- |
|  | **No. of cases (1080)** | **No. of controls (4704)** | **Adjusted OR^b^ (95% CI)** |  | **No. of cases (596)** | **No. of controls (3246)** | **Adjusted OR^b^ (95% CI)** |  |
| **Pre-conception, 0-1 month** | | | | | | | | 0.807 |
| <18 | 227 | 1116 | 1.00 (ref) | <19 | 144 | 792 | 1.00 (ref) |  |
| 18 to <29 | 252 | 1219 | 1.03 (0.83-1.27) | 19 to <29 | 115 | 787 | 0.69 (0.51-0.94) |  |
| 29 to <50 | 249 | 1172 | 1.15 (0.89-1.48) | 29 to <53 | 153 | 835 | 0.99 (0.70-1.40) |  |
| ≥50 | 352 | 1197 | 1.84 (1.38-2.45) | ≥53 | 184 | 832 | 1.51 (0.99-2.30) |  |
| Per 1-SD increase |  |  | 1.25 (1.09-1.42) | Per 1-SD increase |  |  | 1.22 (1.01-1.48) |  |
| Per 10 μg/m^3^ increase |  |  | 1.05 (1.02-1.08) | Per 10 μg/m^3^ increase |  |  | 1.04 (1.00-1.08) |  |
| **Pre-conception, 1-2 months** | | | | | | | | 0.007 |
| <19 | 228 | 1038 | 1.00 (ref) | <21 | 168 | 783 | 1.00 (ref) |  |
| 19 to <31 | 278 | 1277 | 1.14 (0.92-1.40) | 21 to <31 | 99 | 840 | 0.45 (0.33-0.62) |  |
| 31 to <54 | 256 | 1186 | 1.45 (1.13-1.87) | 31 to <58 | 151 | 803 | 0.94 (0.67-1.31) |  |
| ≥54 | 318 | 1203 | 2.20 (1.60-3.02) | ≥58 | 178 | 820 | 1.11 (0.72-1.72) |  |
| Per 1-SD increase |  |  | 1.26 (1.11-1.43) | Per 1-SD increase |  |  | 1.17 (0.99-1.39) |  |
| Per 10 μg/m^3^ increase |  |  | 1.05 (1.02-1.08) | Per 10 μg/m^3^ increase |  |  | 1.03 (1.00-1.07) |  |
| **Pre-conception, 2-3 months** | | | | | | | | 0.035 |
| <23 | 325 | 1053 | 1.00 (ref) | <23 | 186 | 675 | 1.00 (ref) |  |
| 23 to <35 | 246 | 1265 | 0.73 (0.59-0.90) | 23 to <35 | 117 | 921 | 0.41 (0.30-0.56) |  |
| 35 to <67 | 259 | 1154 | 0.94 (0.73-1.22) | 35 to <67 | 129 | 805 | 0.58 (0.40-0.82) |  |
| ≥67 | 250 | 1232 | 0.93 (0.67-1.28) | ≥67 | 164 | 845 | 0.82 (0.53-1.29) |  |
| Per 1-SD increase |  |  | 1.03 (0.89-1.18) | Per 1-SD increase |  |  | 1.15 (0.95-1.38) |  |
| Per 10 μg/m^3^ increase |  |  | 1.01 (0.98-1.04) | Per 10 μg/m^3^ increase |  |  | 1.03 (0.99-1.07) |  |
| **Pre-conception, 0-3 months** | | | | | | | | 0.151 |
| <23 | 297 | 1157 | 1.00 (ref) | <23 | 169 | 743 | 1.00 (ref) |  |
| 23 to <34 | 235 | 1224 | 1.01 (0.82-1.26) | 23 to <35 | 115 | 959 | 0.57 (0.41-0.77) |  |
| 34 to <57 | 241 | 1111 | 1.33 (1.01-1.75) | 35 to <62 | 158 | 724 | 1.18 (0.79-1.74) |  |
| ≥57 | 307 | 1212 | 2.29 (1.59-3.29) | ≥62 | 154 | 820 | 1.31 (0.78-2.20) |  |
| Per 1-SD increase |  |  | 1.35 (1.17-1.57) | Per 1-SD increase |  |  | 1.37 (1.12-1.68) |  |
| Per 10 μg/m^3^ increase |  |  | 1.08 (1.04-1.12) | Per 10 μg/m^3^ increase |  |  | 1.08 (1.03-1.13) |  |
| **Post-conception, 0–1 month** | | | | | | | | 0.406 |
| <17 | 206 | 1088 | 1.00 (ref) | <17 | 116 | 808 | 1.00 (ref) |  |
| 17 to <28 | 254 | 1193 | 1.11 (0.90-1.38) | 17 to <29 | 155 | 777 | 1.22 (0.90-1.64) |  |
| 28 to <52 | 295 | 1238 | 1.28 (1.00-1.63) | 29 to <53 | 148 | 831 | 1.19 (0.83-1.70) |  |
| ≥52 | 325 | 1185 | 1.61 (1.18-2.21) | ≥53 | 177 | 830 | 1.79 (1.14-2.81) |  |
| Per 1-SD increase |  |  | 1.17 (1.01-1.34) | Per 1-SD increase |  |  | 1.08 (0.88-1.33) |  |
| Per 10 μg/m^3^ increase |  |  | 1.04 (1.00-1.07) | Per 10 μg/m^3^ increase |  |  | 1.02 (0.98-1.06) |  |
| **Post-conception, 1–2 months** | | | | | | | | 0.568 |
| <17 | 206 | 1142 | 1.00 (ref) | <16 | 101 | 730 | 1.00 (ref) |  |
| 17 to <29 | 272 | 1153 | 1.29 (1.04-1.60) | 16 to <30 | 177 | 886 | 1.33 (0.98-1.81) |  |
| 29 to <55 | 282 | 1228 | 1.30 (1.01-1.67) | 30 to <65 | 161 | 807 | 1.35 (0.93-1.96) |  |
| ≥55 | 320 | 1181 | 1.36 (0.97-1.90) | ≥65 | 157 | 823 | 1.46 (0.90-2.37) |  |
| Per 1-SD increase |  |  | 1.03 (0.90-1.18) | Per 1-SD increase |  |  | 1.03 (0.85-1.25) |  |
| Per 10 μg/m^3^ increase |  |  | 1.01 (0.98-1.04) | Per 10 μg/m^3^ increase |  |  | 1.01 (0.97-1.05) |  |
| **Post-conception, 2–3 months** | | | | | | | | 0.202 |
| <18 | 227 | 1140 | 1.00 (ref) | <18 | 120 | 792 | 1.00 (ref) |  |
| 18 to <32 | 270 | 1167 | 1.14 (0.92-1.41) | 18 to <33 | 172 | 827 | 1.18 (0.87-1.60) |  |
| 32 to <65 | 310 | 1219 | 1.24 (0.96-1.61) | 33 to <68 | 170 | 797 | 1.58 (1.10-2.28) |  |
| ≥65 | 273 | 1178 | 0.97 (0.69-1.36) | ≥68 | 134 | 830 | 1.27 (0.78-2.05) |  |
| Per 1-SD increase |  |  | 1.01 (0.88-1.17) | Per 1-SD increase |  |  | 0.98 (0.79-1.20) |  |
| Per 10 μg/m^3^ increase |  |  | 1.00 (0.97-1.03) | Per 10 μg/m^3^ increase |  |  | 1.00 (0.95-1.04) |  |
| **Post-conception, 0–3 months** | | | | | | | | 0.189 |
| <21 | 243 | 1191 | 1.00 (ref) | <21 | 135 | 811 | 1.00 (ref) |  |
| 21 to <30 | 220 | 1238 | 1.05 (0.84-1.31) | 21 to <31 | 127 | 804 | 1.05 (0.77-1.44) |  |
| 30 to <63 | 340 | 1098 | 1.67 (1.28-2.17) | 31 to <67 | 199 | 757 | 1.57 (1.07-2.30) |  |
| ≥63 | 277 | 1177 | 1.78 (1.25-2.55) | ≥67 | 135 | 874 | 1.38 (0.81-2.35) |  |
| Per 1-SD increase |  |  | 1.19 (1.01-1.40) | Per 1-SD increase |  |  | 1.16 (0.92-1.47) |  |
| Per 10 μg/m^3^ increase |  |  | 1.04 (1.00-1.09) | Per 10 μg/m^3^ increase |  |  | 1.03 (0.98-1.09) |  |

Abbreviations: CI, confidence interval; OR, odds ratios; SD, standard deviation; SO_2_,sulfur dioxide; ref, reference.

^a^ SO_2_ concentrations (μg/m³) are based on the monthly average concentrations, which are then averaged over different exposure windows and analyzed in quartiles (determined from controls).

^b^ Logistic regression analysis adjusting for season of conception, gravidity, parity, maternal education and maternal nitrogen dioxide and particulate matter with an aerodynamic diameter ≤ 10 μm exposures during the same exposure window.

**Supplemental Table S3 Standardized differences for covariates used to estimate propensity scores across two case-control sets. (Association between maternal exposure to SO_2_ and congenital ear malformations in offspring: a population-based case-control study in Liaoning Province, China, 2010–2015)**

| **Characteristics** | **Standardized Difference (%)** | |
| --- | --- | --- |
|  | **Full population (n = 1676)** | **Propensity score-matched subsample (n = 767)** |
| **Season of conception** |  |  |
| Spring | 0.00 | -0.05 |
| Summer | -0.20 | 0.01 |
| Fall | 0.12 | 0.04 |
| Winter | 0.11 | 0.01 |
| **Maternal age, years** | | |
| <30 | 0.11 | -0.07 |
| ≥30 | -0.11 | 0.07 |
| **Gravidity** | | |
| 1 | -0.17 | -0.08 |
| ≥2 | 0.17 | 0.08 |
| **Parity** | | |
| 1 | -0.53 | 0.01 |
| ≥2 | 0.53 | -0.01 |
| **Maternal education** | | |
| Elementary school or less | 0.05 | -0.10 |
| Middle school | 0.06 | 0.01 |
| High school | 0.04 | 0.03 |
| College or above | -0.12 | 0.00 |

**Supplemental Table S4 Association of exposure to ambient SO_2_ during different gestation periods and congenital ear malformations in the propensity score-matched subsample. (Association between maternal exposure to SO_2_ and congenital ear malformations in offspring: a population-based case-control study in Liaoning Province, China, 2010–2015)**

| **Quartile of SO_2_ level^a^** | **No. of**  **cases** | **No. of**  **controls** | **Adjusted OR^b^ (95% CI)** | **Quartile of SO_2_ level^a^** | **No. of**  **cases** | **No. of**  **controls** | **Adjusted OR^b^ (95% CI)** |
| --- | --- | --- | --- | --- | --- | --- | --- |
| **Pre-conception, 0-1 month** | | | | **Pre-conception, 1-2 months** | | | |
| <23 | 256 | 135 | 1.00 (ref) | <23 | 248 | 124 | 1.00 (ref) |
| 23 to <32 | 120 | 240 | 0.52 (0.37-0.72) | 23 to <35 | 186 | 259 | 0.65 (0.47-0.89) |
| 32 to <68 | 215 | 200 | 1.76 (1.24-2.50) | 35 to <79 | 205 | 186 | 1.46 (1.02-2.09) |
| ≥68 | 176 | 192 | 3.62 (2.30-5.76) | ≥79 | 128 | 198 | 2.25 (1.38-3.68) |
| Per 1-SD increase |  |  | 1.84 (1.52-2.23) | Per 1-SD increase |  |  | 1.83 (1.51-2.23) |
| Per 10 μg/m^3^ increase |  |  | 1.11 (1.08-1.15) | Per 10 μg/m^3^ increase |  |  | 1.11 (1.08-1.15) |
| **Pre-conception, 2-3 months** | | | | **Pre-conception, 0-3 months** | | | |
| <24 | 264 | 188 | 1.00 (ref) | <25 | 252 | 194 | 1.00 (ref) |
| 24 to <41 | 188 | 165 | 1.21 (0.88-1.67) | 25 to <38 | 166 | 189 | 1.20 (0.87-1.65) |
| 41 to <102 | 230 | 220 | 2.09 (1.51-2.90) | 38 to <79 | 234 | 192 | 2.57 (1.85-3.60) |
| ≥102 | 85 | 194 | 2.28 (1.38-3.79) | ≥79 | 115 | 192 | 3.87 (2.35-6.42) |
| Per 1-SD increase |  |  | 1.55 (1.28-1.87) | Per 1-SD increase |  |  | 1.97 (1.59-2.44) |
| Per 10 μg/m^3^ increase |  |  | 1.08 (1.05-1.12) | Per 10 μg/m^3^ increase |  |  | 1.15 (1.10-1.20) |
| **Post-conception, 0–1 month** | | | | **Post-conception, 1–2 months** | | | |
| <23 | 266 | 145 | 1.00 (ref) | <23 | 252 | 162 | 1.00 (ref) |
| 23 to <32 | 115 | 235 | 0.50 (0.36-0.69) | 23 to <32 | 128 | 218 | 0.59 (0.43-0.82) |
| 32 to <66 | 194 | 188 | 1.83 (1.28-2.62) | 32 to <82 | 262 | 193 | 2.22 (1.58-3.12) |
| ≥66 | 192 | 199 | 3.31 (2.18-5.06) | ≥82 | 125 | 194 | 2.41 (1.50-3.91) |
| Per 1-SD increase |  |  | 1.85 (1.54-2.21) | Per 1-SD increase |  |  | 1.76 (1.47-2.12) |
| Per 10 μg/m^3^ increase |  |  | 1.12 (1.08-1.16) | Per 10 μg/m^3^ increase |  |  | 1.12 (1.08-1.16) |
| **Post-conception, 2–3 months** | | | | **Post-conception, 0–3 months** | | | |
| <23 | 224 | 161 | 1.00 (ref) | <24 | 235 | 162 | 1.00 (ref) |
| 23 to <32 | 128 | 198 | 0.66 (0.48-0.92) | 24 to <34 | 140 | 244 | 0.87 (0.62-1.21) |
| 32 to <79 | 284 | 216 | 2.33 (1.67-3.27) | 34 to <76 | 251 | 169 | 3.16 (2.22-4.51) |
| ≥79 | 131 | 192 | 2.69 (1.69-4.32) | ≥76 | 141 | 192 | 3.89 (2.34-6.52) |
| Per 1-SD increase |  |  | 1.76 (1.47-2.10) | Per 1-SD increase |  |  | 2.23 (1.81-2.76) |
| Per 10 μg/m^3^ increase |  |  | 1.12 (1.08-1.16) | Per 10 μg/m^3^ increase |  |  | 1.19 (1.14-1.25) |

Abbreviations: CI, confidence interval; OR, odds ratios; SD, standard deviation; SO_2_,sulfur dioxide; ref, reference.

^a^ SO_2_ concentrations (μg/m³) are based on the monthly average concentrations, which are then averaged over different exposure windows and analyzed in quartiles (determined from controls).

^b^The analysis was matched on maternal age, season of conception, gravidity, parity and maternal education. Additional adjustment was made for nitrogen dioxide and particulate matter with an aerodynamic diameter ≤ 10 μm exposure levels during the same period.

**Supplemental Figure S1 Monthly mean SO_2_ concentrations from 2010 to 2015, by study site. (Association between maternal exposure to SO_2_ and congenital ear malformations in offspring: a population-based case-control study in Liaoning Province, China, 2010–2015
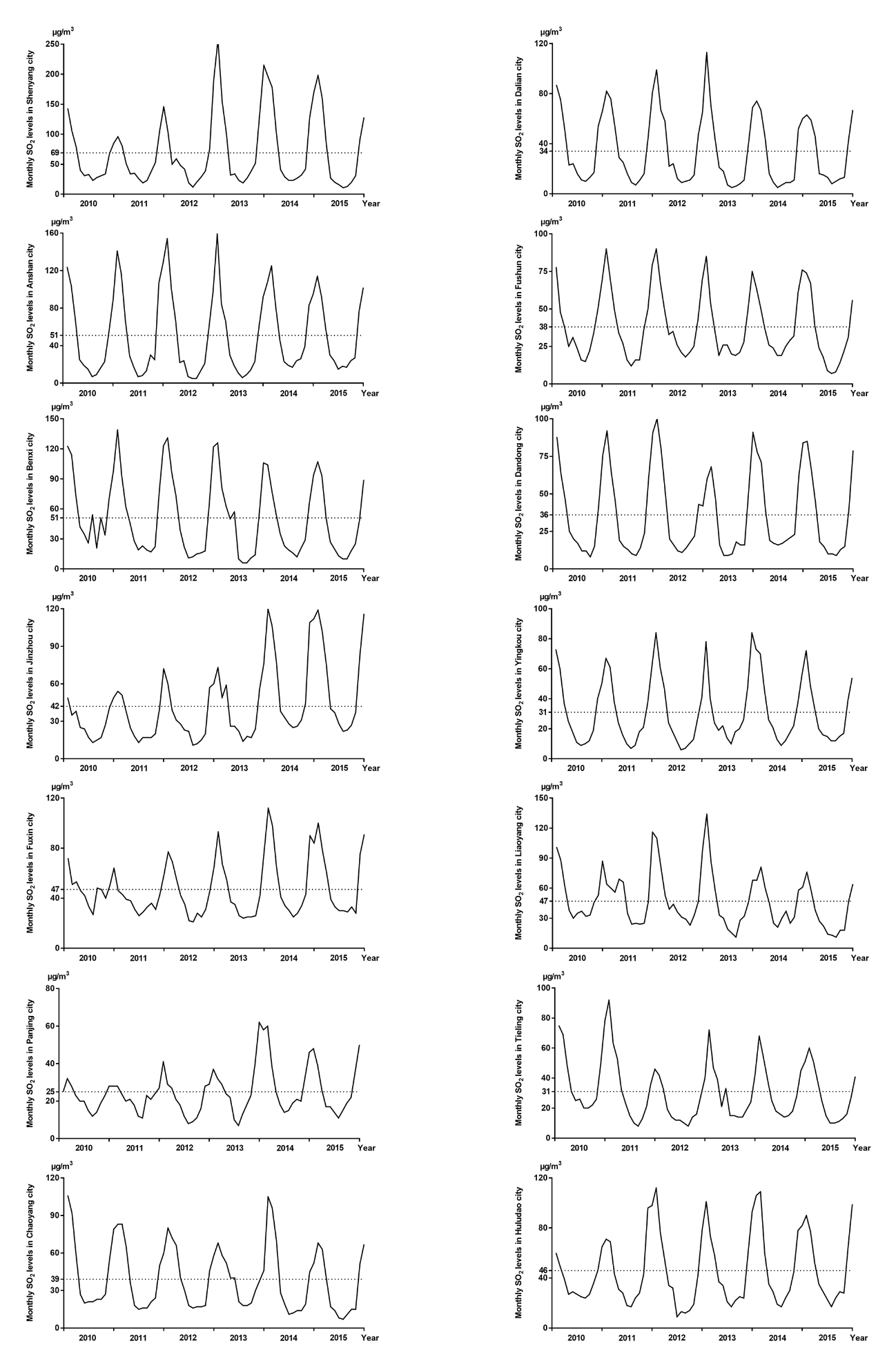
)**
